# Medical Convention of Texas

**Published:** 1877-05-20

**Authors:** 


### MEDICAL CONVENTION OF TEXAS.

The ninth annual session of the Medical Convention of the State of Texas assembled at Galveston, on the 9th ult., and was called to order by the President, Dr. R. H. Harrison. The roll was called by Dr. East, the Secretary, and a quorum found to be present. A very appropriate and cordial address of welcome was made by Prof. Greenville Dowel, in which he remarks of Galveston: "Twelve years ago we had 13,000 inhabitants, and 36 physicians, now we have 40,000 inhabitants, and not so much to do as then." A result which he attributes to drainage and sanitary improvements.

A resolution endorsing the action of the Travis County Medical Society, in refusing to treat with irregular practitioners, gave rise to a somewhat prolonged and heated discussion. A certain member of the convention having acted upon a State Medical Examining Board, composed in part of irregulars. The Travis County Society was finally sustained, but the offending brother was exhonorated from blame.

A sumptuous banquet was provided for the members, to which an address was delivered by Dr. Kelly, President of the State Association.

Several interesting papers and reports were read in the subsequent proceedings of the body which we cannot notice at present.

